# Effects of Chirality on the Antifungal Potency of Methylated Succinimides Obtained by *Aspergillus fumigatus* Biotransformations. Comparison with Racemic Ones

**DOI:** 10.3390/molecules18055669

**Published:** 2013-05-15

**Authors:** Maximiliano Sortino, Agustina Postigo, Susana Zacchino

**Affiliations:** Pharmacognosy Area, Faculty of Biochemical and Pharmaceutical Sciences, National University of Rosario, Suipacha 531, 2000-Rosario, Argentina; E-Mails: msortino@fbioyf.unr.edu.ar (M.S.); aguspostigo@hotmail.com (A.P.)

**Keywords:** biotransformation, *Aspergillus fumigatus*, enantioselective reduction, enhanced antifungal activity, chiral succinimides, methylated succinimides

## Abstract

Eighteen (3*R*) and (3*R*,4*R*)-*N*-phenyl-, *N*-phenylalkyl and *N-*arylsuccinimides were prepared with high enantioselectivity by biotransformation of maleimides with *A. fumigatus*. This environmentally friendly, clean and economical procedure was performed by the whole-cell fungal bioconversion methodology. Their corresponding eighteen racemic succinimides were prepared instead by synthetic methods. Both, the racemic and the chiral succinimides were tested simultaneously by the microbroth dilution method of CLSI against a panel of human opportunistic pathogenic fungi of clinical importance. Chiral succinimides showed higher antifungal activity than the corresponding racemic ones and the differences in activity were established by statistical methods. The bottlenecks for developing chiral drugs are how to obtain them through a low-cost procedure and with high enantiomeric excess. Results presented here accomplish both these objectives, opening an avenue for the development of asymmetric succinimides as new antifungal drugs for pharmaceutical use.

## 1. Introduction

Chiral succinimides, containing asymmetric carbons (in position 3- or 3,4- of the imido ring) have demonstrated to be core structural units with interesting biological activities. They have shown anxiolytic, antidepressant effects, and the ability to inhibit protein synthesis and human enzymes, such as leucocyte elastase, cathepsin G, proteinase 3 and glycosidase, among others. As a consequence, they have become good clinical drug candidates for several diseases [1,2,3,4,5,6].

Regarding antimicrobial activity, the chiral succinimides andrimid and moraimide B showed potent *in vitro* antibacterial activity against antibiotic-resistant human pathogens as methicillin*-*resistant *Staphylococcus aureus* [7]. In turn, hirsutellones inhibited the growth of *Mycobacterium tuberculosis* [4]. These findings have led to an increased interest in the clinical use of these asymmetric structures as a class of potential antimicrobial agents.

The bottleneck for developing chiral drugs for pharmaceutical use is to obtain them in a cheap and friendly procedure with high enantiomeric excesses (*ees*). Asymmetric synthesis by chemical procedures typically requires the use of expensive catalysts containing transition metal ions [8], which often prevents its commercial development. In contrast, the application of biocatalysts using whole cells in their native forms in aqueous/organic media has shown to be a highly selective, environmentally safe and cost effective method of producing enantiomeric compounds [9,10,11,12].

**Figure 1:**
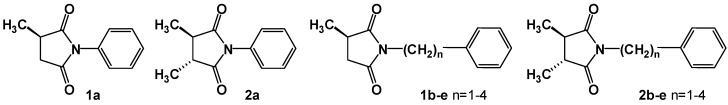
Structures of (3*R*)-(+)-methyl-*N-*phenylsuccinimide **1a**, (3*R*,4*R*)-(+)-dimethyl-*N-*phenylsuccinimide **2a**, (3*R*)-(+)-methyl-*N-*phenylalkylsuccinimides **1b**–**e** and (3*R*,4*R*)-(+)-dimethyl-*N-*phenylalkylsuccinimides **2b**–**e** [alkyl chain = (CH_2_)_n_ (n = 1–4)].

In the course of our project aimed at generating new chiral compounds through fungal biotransformations, we previously reported the preparation of (3*R*)-(+)-methyl-*N-*phenylsuccinimide (**1a**) and (3*R*,4*R*)-(+)-dimethyl-*N-*phenylsuccinimide (**2a**). These compounds were obtained in >99% *ee* by bioconversion of 3-methyl-*N-*phenyl- and 3,4-dimethyl-*N-*phenylmaleimide with *Aspergillus fumigatus* ATCC 26934, which proved to be the most effective catalyst among the fifteen strains tested [13]. In a subsequent paper, we reported the production of (3*R*)-(+)-methyl-*N-*phenylalkylsuccinimides **1b**–**e** and (3*R*,4*R*)-(+)-dimethyl-*N-*phenylalkylsuccinimides **2b**–**e** [alkyl chain = (CH_2_)_n_ (n = 1–4)] (Figure 1) with excellent enantioselectivities (>99% *ee*) from 3-methyl- and 3,4-dimethyl-*N-*phenyl-alkylmaleimides, with the same *A. fumigatus* strain [14].

**Scheme 1:**
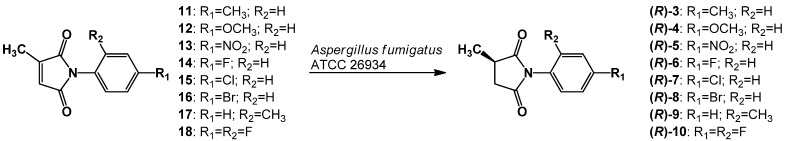
Biotransformation of 3-methyl-*N-*arylmaleimides **11**–**18** to (3*R*)-(+)-methyl-*N-*arylsuccinimide **3**–**10** with *Aspergillus fumigatus* ATCC 26934.

This prompted us to expand the knowledge on the ability of *A. fumigatus* to stereoselectively hydrogenate eight related 3-methyl-*N*-arylmaleimides to produce asymmetric succinimides **3**–**10** (Scheme 1). These compounds possess a substituted benzene ring with either electron-withdrawing or electron-donor groups on its *p*- or *o*-positions. It is known that the application of the same microorganism on different substrates does not always result in similar transformations or enantioselectivities of catalyzed reactions.

Chiral compounds **3**–**10**, along with the previously obtained chiral **1a**–**e** and **2a**–**e**, were tested here for antifungal properties against a panel of human opportunistic pathogenic fungi using standardized procedures. In addition, racemic succinimides (±)-**1a**–**e**; **-2a**–**e**; **-3**–**10** were synthesized and tested simultaneously against the same panel of fungi in order to compare their antifungal activities. It is well known that isomers can differ in their biological activities, thus the knowledge of the properties of both, racemic and enantiomeric forms of a compound is of great significance in the pharmaceutical field, leading to a better understanding of the concentration-effect relationships, adverse effects, activity or toxicity [15].

## 2. Results and Discussion

For the sake of simplicity, the thirty six compounds: racemic (*rac*)- and chiral **1a**–**e**, **2a**–**e**, **3**–**10**, were grouped into three classes: (A) (*rac*)- and (3*R*)-methyl-*N-*phenyl- and *N-*phenylalkyl-succinimides **1a**-**1e**; (B) (*rac*)- and (3*R*,4*R*)-dimethyl-*N-*phenyl- and *N-*phenylalkyl- succinimides **2a**–**2e**; and (C) (*rac*)- and (3*R*)-methyl-*N-*arylsuccinimides **3**–**10**.

As stated above, (3*R*)- and (3*R*,4*R*)-succinimides of groups (A) and (B) (compounds **1a**–**e** and **2a**–**e**) were previously obtained with >99% *ee* by fungal biotransformation of the respective maleimides with *A. fumigatus* ATCC 26934 [13,14,16]. In turn, racemic ones were obtained by catalytic hydrogenation of the respective maleimides, as previously reported [14].

Chiral compounds of group C [(*3R*)–**3**–**10**)], on the other hand, were obtained by submitting the respective maleimides **11**–**18** to biotransformation with the same strain of *A. fumigatus* used for obtaining chiral succinimides of groups (A) and (B) (Scheme 1). (*Rac*)-**3**–**10** were obtained by catalytic hydrogenation with H_2_ (Pd/C). In turn, maleimides **11**–**18** were obtained following reported procedures [14].

**Scheme 2:**
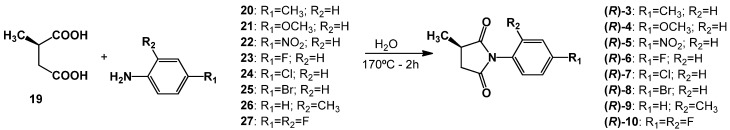
Synthesis of (3*R*)-methyl-*N-*aryl-succinimides **3**–**10** from (2*R*)-methylsuccinic acid **19** and respective anilines **20**–**27**.

The structures of **3**–**10** were corroborated by MS and ^1^H- and ^13^C-NMR. To assist in the assignment of both the absolute configuration and the *ee* of each chiral succinimide, *R*-enantiomers of **3**–**10** were also synthesized from (*R*)-2-methylsuccinic acid **19** and the respective anilines **20**–**27** (Scheme 2) [17]. Synthetic (3*R*)-**3**-**10** were all dextrorotatory, therefore indicating that the biotransformation products (+)-**3**-**10** had the *R*-configuration.

Chiral GC of *rac*- and (3*R*)-(**3**-**10**) allowed us to determine that (3*R*)-enantiomers **3**-**10** eluted before the *S*-ones. These data were used to determine the *ee* of each biotransformation mixture and to confirm its absolute configuration. Results showed that **11**-**18** were converted into (3*R*)-(+)-**3**-**10** with *ee* ≥ 98%. Table 1 shows % conversion; % *ee* and absolute configurations of **3**-**10** [data of succinimide *R*-**1a**, obtained from 3-methyl-*N*-phenylmaleimide (**28**) [13], were also included].

**Table 1:**
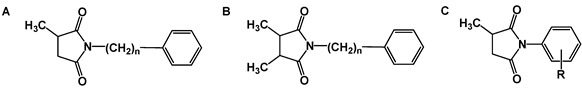
Biotransformation of 3-methyl-*N-*arylmaleimides **11**–**18** to (3*R*)-(+)-methyl-*N-*arylsuccinimides **3**–**10**.

Substrate	R_1_	R_2_	Product	% *ee*	% Conv.
11	CH_3_	H	(*R*)-(+)-3	97	93
12	OCH_3_	H	(*R*)-(+)-4	>99	97
13	NO_2_	H	(*R*)-(+)-5	98	95
14	F	H	(*R*)-(+)-6	98	99
15	Cl	H	(*R*)-(+)-7	97	91
16	Br	H	(*R*)-(+)-8	98	81
17	H	CH_3_	(*R*)-(+)-9	98	65
18	F	F	(*R*)-(+)-10	99	55
28 *	H	H	(*R*)-(+)-1a	>99	99

* Results previously reported [13]; % *ee*: % enantiomeric excess (calculated by chiral GC); % Conv: Conversion percentages (determined by GC analysis by using the TIC (total ion current) with the following equation: % conversion: product TIC/(product TIC + substrate TIC) × 100.

The above results expand the knowledge on the ability of *A. fumigatus* ATCC 26934 to enantioselectively hydrogenate eight related prochiral 3-methyl-N-arylmaleimides **11**–**18** to produce chiral succinimides (3*R*)-**3**–**10** with high enantioselectivity and in high yields. The fungus showed the same enantioface preference, irrespective of the substituent on the benzene ring which was the same results that has been observed with their analogues **1a**–**e** and **2a**–**e** [13,14]. To our knowledge, there are no previous reports on chiral synthesis of (3*R*)-methyl-*N*-arylsuccinimides **3**, **5**-**10** by any of the chemical or enzymatic methods. Instead, (3*R*)-(+)-**4** has been previously obtained by biotransformation with the plant *Marchantia polymorpha* [18].

Compounds (3*R*)-(**3**–**10)**, along with the previously obtained chiral compounds **1a**–**e** and **2a**–**e**, were tested for antifungal properties against a panel of eleven human opportunistic pathogenic fungi comprising yeasts (*Candida* spp., *Cryptococcus neoformans*, *Saccharomyces cerevisiae* and the dermatophytes *Microsporum gypseum*, *Trichophyton rubrum* and *Trichophyton mentagrophytes*). The selection of these species was due to their high clinical incidence mainly among immunocompromised patients. Thus, species of the genus *Candida* are among the leading causes of nosocomial, blood stream infections worldwide and, although *C. albicans* was in the past the usual species associated with invasive infections, at present non*-albicans Candida* spp. (*C. tropicalis*, *C. glabrata*, *C. parapsilopsis*, *C. krusei* and*C. lusitaneae*) comprise more than half of human candidiasis isolates [19].

In turn, *C. neoformans* was selected because it remains an important life-threatening species for immunocompromised hosts, particularly for patients infected with HIV and therefore, new compounds that act against this fungus are highly welcome [20,21]. Regarding dermatophytes of the genus *Microsporum* and *Trichophyton*, they were selected because both genera are the cause of approximately 80–93% of chronic and recurrent human superficial infections which, although not life-threatening, diminish the quality of life of patients because they are difficult to eradicate [22].

To determine the Minimum Inhibitory Concentration (MIC), amounts of compounds from 250 μg·mL^−1^ were incorporated into growth media according to the CLSI standardized procedures [23,24]. Amphotericin B, terbinafine, and ketoconazole were used as positive controls. The Enhancement Ratio (ER), which is a measure of how many-fold the MIC was reduced in each enantiomeric compound compared to its corresponding racemic one, was calculated as the ratio between MIC (*rac*)/MIC (enantiomer). Results are shown in Table 2.

**Table 2:**
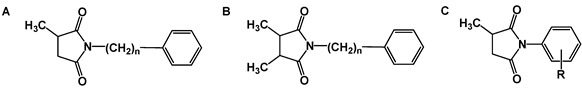
Antifungal activity (MICs in μg·mL^−1^) of 3-methyl-*N-*phenyl- or *N-*phenylalkylsuccinimides **1a**–**e** (**A**); 3,4-dimethyl-*N-*phenyl- or phenylalkylsuccinimides **2a**–**e** (**B**); and 3-methyl-*N-*arylsuccinimides **3**–**10** (**C**), in their racemic and enantiomeric forms against a panel of yeasts and dermatophytes.

		Type		n	*Conf.*	*Ca* ^1^	*Ct* ^2^	*Ck* ^3^	*Cg* ^4^	*Cp* ^5^	*Cl* ^6^	*Sc* ^7^	*Cn* ^8^	*Mg* ^9^	*Tr* ^10^	*Tm* ^11^
**1**	**1a**	A	0	-	*R-*	62.5	62.5	62.5	62.5	62.5	62.5	62.5	62.5	62.5	31.3	62.5
					*rac-*	125	125	125	125	125	125	125	125	62.5	125	62.5
					ER	2	2	2	2	2	2	2	2	-	4	-
	**1b**	A	1	-	*R-*	15.6	31.3	62.5	62.5	62.5	31.3	62.5	15.6	125	62.5	62.5
					*rac-*	62.5	125	125	125	125	125	125	62.5	250	125	250
					ER	4	4	2	2	2	4	2	4	2	2	4
	**1c**	A	2	-	*R-*	31.3	62.5	125	62.5	31.3	62.5	62.5	62.5	125	62.5	31.3
					*rac-*	125	125	125	125	125	125	125	125	125	62.5	62.5
					ER	4	2	-	2	4	2	2	2	-	-	2
	**1d**	A	3	-	*R-*	62.5	125	125	62.5	125	125	125	62.5	62.5	125	62.5
					*rac-*	125	125	125	125	125	125	125	125	125	250	250
					ER	2	-	-	-	-	-	-	2	2	2	4
	**1e**	A	4	-	*R-*	31.3	31.3	62.5	125	62.5	125	62.5	31.3	125	62.5	31.3
					*rac-*	62.5	125	125	125	125	125	125	125	250	125	125
					ER	2	4	2	-	2	-	2	4	2	2	4
**2**	**2a**	B	0	-	3*R,*4*R-*	31.3	31.3	62.5	62.5	31.3	62.5	62.5	62.5	125	125	62.5
					*rac-*	125	62.5	62.5	125	62.5	62.5	125	125	125	125	125
					ER	4	2	-	2	2	-	2	2	-	-	2
	**2b**	B	1	-	3*R,*4*R-*	31.3	62.5	62.5	125	62.5	62.5	125	62.5	62.5	62.5	31.3
					*rac-*	125	125	125	125	125	125	125	125	62.5	62.5	125
					ER	4	2	2	-	2	2	-	2	-	-	4
	**2c**	B	2	-	3*R,*4*R-*	15.6	125	125	62.5	62.5	62.5	125	62.5	125	125	62.5
					*rac-*	125	125	250	125	250	125	125	125	250	250	62.5
					ER	8	-	2	2	4	2	-	2	2	2	-
	**2d**	B	3	-	3*R,*4*R-*	31.3	62.5	62.5	62.5	62.5	62.5	62.5	62.5	62.5	31.3	31.3
					*rac-*	62.5	125	125	125	125	250	125	125	125	62.5	125
					ER	2	2	2	2	2	4	2	2	2	2	4
	**2e**	B	4	-	3*R,*4*R-*	62.5	62.5	62.5	62.5	62.5	62.5	62.5	31.3	62.5	62.5	62.5
					*rac-*	125	125	62.5	125	125	125	125	125	125	125	125
					ER	2	2	-	2	2	2	2	4	2	2	2
**3**		C	0	4'-CH_3_	*R-*	62.5	62.5	62.5	62.5	62.5	62.5	62.5	62.5	125	125	125
					*rac-*	250	250	125	125	250	250	125	62.5	250	250	250
					ER	4	4	2	2	4	4	2	-	2	2	2
**4**		C	0	4'-OMe	*R-*	125	62.5	62.5	62.5	62.5	62.5	125	62.5	125	125	125
					*rac-*	250	125	125	125	125	125	125	62.5	250	250	250
					ER	2	2	2	2	2	2	-	-	2	2	2
**5**		C	0	4'-NO_2_	*R-*	62.5	125	62.5	125	62.5	125	125	62.5	125	125	125
					*rac-*	250	125	125	125	125	125	125	125	250	250	250
					ER	4	-	2	-	2	-	-	2	2	2	2
**6**		C	0	4'-F	*R-*	62.5	62.5	31.3	62.5	31.3	62.5	62.5	62.5	62.5	62.5	62.5
					*rac-*	250	250	125	125	250	250	125	62.5	125	125	125
					ER	4	4	4	2	8	4	2	-	2	2	2
**7**		C	0	4'-Cl	*R-*	125	62.5	125	62.5	125	62.5	62.5	31.3	125	125	125
					*rac-*	250	250	125	250	250	250	125	125	250	250	250
					ER	2	4	-	4	2	4	2	4	2	2	2
**8**		C	0	4'-Br	*R-*	125	62.5	62.5	62.5	62.5	62.5	62.5	62.5	62.5	125	125
					*rac-*	250	250	125	125	250	250	125	125	125	250	125
					ER	2	4	2	2	4	4	2	2	2	2	-
**9**		C	0	2'-CH_3_	*R-*	125	125	62.5	125	125	125	125	62.5	125	125	125
					*rac-*	250	125	125	125	250	125	125	125	250	250	250
					ER	2	-	2	-	2	-	-	2	2	2	2
**10**		C	0	2',4'-F_2_	*R-*	62.5	62.5	31.3	62.5	31.3	125	62.5	62.5	62.5	62.5	125
					*rac-*	250	250	125	125	250	250	125	125	125	250	250
					ER	4	4	4	2	8	2	2	2	2	4	2
**AmphotericinB**						0.97	0.48	0.49	0.49	0.98	0.98	0.48	0.24	0.12	0.06	0.06
**Ketoconazole**						0.48	0.12	62.5	1.95	0.98	0.98	0.48	0.24	0.06	0.03	0.03
**Terbinafine**						-	-	-	-	-	-	-	-	0.03	0.01	0.03

1: *Candida albicans* ATCC 10231, 2: *C. tropicalis* CCC 131-2000: *C. krusei* CCC 117-2000, 4: *C. glabrata* CCC 115-2000, 5: *C. parapsilosis* CCC 124-2000, 6: *C. lusitaniae* CCC 134-2000, 7: *Saccharomyces cerevisiae* ATCC 9763, 8: *Cryptococcus neoformans* ATCC 32264, 9: *Microsporum gypseum* CCC 115-2000, 10: *Trichophyton rubrum* CCC 113 2000, 11: *T. mentagrophytes* ATCC 9972. CCC = Colección de Cultivos del CEREMIC (Centro de Referencia en Micología). ATCC: American Type Culture Collection. Conf: configuration; ER = MIC (*rac*)/MIC (*R* form).

The comparison of the 198 MICs of racemic mixtures (18 compounds x 11 fungi) with the same number of MICs of enantiomers, allowed us to detect that 158 MICs of chiral forms (80%) were statistically significantly lower (*p* < 0.05) than the corresponding racemic form ones. Of them, 117 MICs were two orders of magnitude lower (ER = 2); 38 MICs were four-fold lower (ER = 4), and three MICs were eight-fold lower (ER = 8). *C. albicans* was shown to be the most sensitive species to chirality, showing enhancements in all pairs of compounds tested. The statistical analyses were performed by the non*-*parametric ANOVA, Kruskal-Wallis test followed by Dunn’s multiple comparisons and Wilcoxon’s signed rank test (*p* < 0.05).

**Figure 2:**
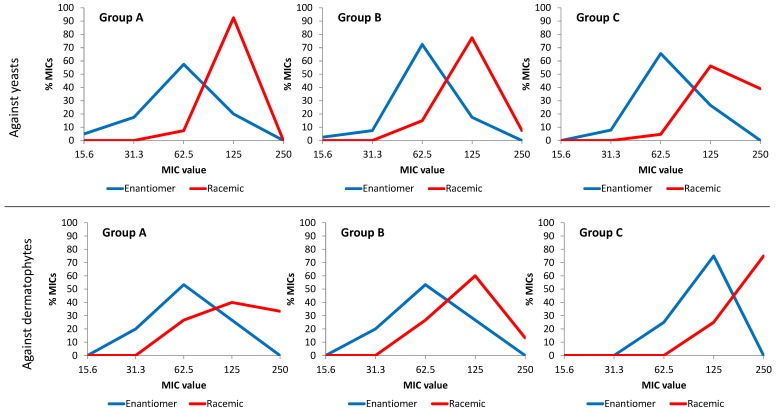
Percentages of the different MIC values (number of MICs at each concentration/total number of MICs) × 100, for the enantiomeric (3*R*) or (3*R*,4*R*) and racemic forms of succinimides of either group (A), (B), or (C) acting against yeasts and dermatophytes.

This overall trend of a better antifungal behavior of (3*R*)- and (3*R*,4*R*)- forms, with respect to racemic ones against the whole panel of fungi, was analyzed within each group of compounds for each type of fungus (yeasts and dermatophytes) (Figure 2). Each graph shows the percentage of MICs of each value, calculated as follows: [number of MICs of each value (250, 125, 62.5, 31.3 and 15.6 µg·mL^−1^) × 100/total number of MICs] displayed by enantiomeric forms and by racemic mixtures within each group of compounds. The associations between MIC values and enantiomeric or racemic forms within each group and type of fungi for the different groups were established with the Score’s test (*p* < 0.05) [25].

**Figure 3:**
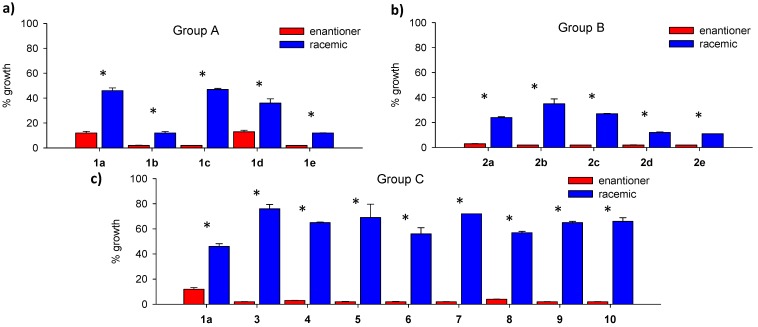
Comparative values of the inhibitory activities of antifungal succinimides in their enantiomeric and racemic forms (at 125 μg/mL) expressed as percentage of growth against *Candida albicans* ATCC 10231. * *p* < 0.05.

To corroborate the above findings from another point of view, we compared the percentages of fungal growth inhibition for the enantiomeric form and the racemic mixture of each compound, at a fixed concentration. Figure 3 shows the comparative antifungal inhibitory activities of chiral *vs* racemic forms of each of the eighteen structures tested at 125 µg·mL^−1^ as a measure of % of growth of *C. albicans.*

It can be observed in the three groups, that each enantiomer showed a significantly lower percent of growth that its respective racemic form, confirming the previous analyses. These comparisons between groups were performed with Student’s *t* test.

It is worth noting that the overall antifungal behavior of chiral forms was better than that of racemic ones, irrespective of the succinimides’ *N*-substituents, since chiral succinimides with or without an alkyl chain between the *N* atom and the phenyl group, or with or without substituents in the 2' and 4'-positions of the benzene ring, showed better antifungal activity than their corresponding racemic forms.

## 3. Experimental

### 3.1. General

Solvents and reagents were purchased from Sigma-Aldrich (St. Louis, MO, USA) and were purified in the usual manner. ^1^H- and ^13^C-NMR spectra were recorded on a Bruker (Karlsruhe, Germany) 300 MHz NMR spectrometer. Compounds were dissolved in deuterated solvents from commercial sources (Sigma-Aldrich) with tetramethylsilane (TMS) as the internal standard. Chemical shifts (δ) are reported in ppm relative to the solvent peak (CHCl_3_ in CDCl_3_ at 7.26 ppm for protons and at 77.0 ppm for carbons). Signals are designated as follows: s, singlet; d, doublet; dd, doublets of doublets; t, triplet; m, multiplet; q, quartet. Melting points were obtained on or using an Electrothermal apparatus (Southend-on-Sea, Essex, UK) and are uncorrected. Optical rotations were measured with a Jasco DIB-1000 (Easton, USA) at room temperature. GC analyses were performed on a CG-MS Turbo Mass Perkin Elmer (Waltman, MA, USA), equipped with a fused silica gel column (SE-30 25 m × 0.22 mm I.D.) with He as a carrier gas in a column PE1 30 m × 0.25 mm (I.D.), film 0.1 μm, ionization energy 70 eV with a temperature program of 70–200 °C at 10 °C min^−1^; total time 30 min. Chiral gas chromatograms were obtained with a CG-MS QP2010-Plus (Shimadzu, Kioto, Japan), with He as a carrier gas on a Beta Dex-325 column (30 m × 0.25 mm I.D.), ionization energy 70 eV, with a temperature program of 60–200 °C at 2 °C·min^−1^; total time 80 min.

### 3.2. Synthesis

*3-Methyl-N-arylmaleimides***11**–**18**. The synthesis of maleimides **11**–**18** was performed by mixing an equimolecular amount of substituted anilines **20**–**27** (5 mmol), dissolved in CHCl_3_ (1 mL), and maleic anhydride **29** in CHCl_3_ (5 mL) and stirring during 1 h. The solid which precipitated out of the reaction mixture (maleamic acid) was filtered off. The whole amount of maleamic acid was dissolved in acetic anhydride (5 mL) and sodium acetate (100 mg) was added. The mixture was heated for 2 h under reflux. The reaction was cooled, quenched with water and the aqueous solution was extracted with Et_2_O, dried over Na_2_SO_4_, filtered, and the solvent evaporated. The product was purified by silica gel column chromatography using a mixture of hexane and ethyl acetate (9:1) as eluent. Spectroscopic NMR data of compound **12** was identical to that previously reported [18]. Compounds **11**, **13**–**18** have been previously reported by Chemical Abstract Service (CAS) (**11**: CAS 3120-12-5, **13**: CAS 10490-21-8, **14**: CAS 883033-95-2, **15**: CAS 59648-09-8, **16**: CAS 134939-24-5, **17**: CAS 131406-19-4, **18**: CAS 124704-71-8) but, to the best of our knowledge, complete NMR spectral data were not available in the literature, so below, the complete spectroscopic data of these compounds are described.

*3-Methyl-N-(4'-methylphenyl)maleimide* (**11**). White solid. Mp: 92–95 °C. Yield: 73%. ^1^H-NMR (CDCl_3_): 2.17 (3H, d, *J* = 1.8 Hz, CH_3_); 2.37 (3H, s, CH_3_); 6.46 (1H, q, *J* = 1.8 Hz, H-3); 7.13–7.33 (4H, m, HAr) ppm. ^13^C-NMR (CDCl_3_): 11.2; 21.1; 125.9; 127.4; 128.9; 129.7; 137.8; 145.7; 169.8; 170.8 ppm. MS: *m/z* 201 (M^+^, 100%), 202 (M^+^+1, 13%).

*3-Methyl-N-(4'-methoxyphenyl)maleimide* (**12**). White solid. Mp: 99–101 °C. Yield: 62%. ^1^H-NMR (CDCl_3_): δ 2.17 (3H, d, *J* = 1.8 Hz, CH_3_); 3.83 (3H, s, OCH_3_); 6.46 (1H, q, *J* = 1.8 Hz, H-3); 6.97 (2H, d, *J* = 8.9 Hz, H-3',5'); 7.23 (2H, d, *J* = 8.9 Hz, H-2';6') ppm. ^13^C-NMR (CDCl_3_): 11.2; 55.5; 114.4; 124.3; 127.4; 127.5; 145.7; 159.0; 169.9; 170.9 ppm. MS: *m/z* 217 (M^+^, 100%), 218 (M^+^+1, 13%).

*3-Methyl-N-(4'-nitrophenyl)maleimide* (**13**). White solid. Mp: 126–128 °C. Yield: 51%. ^1^H-NMR (CDCl_3_): 2.11 (3H, d, *J* = 1.8 Hz, CH_3_); 6.48 (1H, q, *J* = 1.8 Hz, H-3); 7.62 (2H, d, *J* = 9.3 Hz, H-2',6'); 8.22 (2H, d, *J* = 9.3 Hz, H-3',5') ppm. ^13^C-NMR (CDCl_3_): 11.2; 124.4; 125.2; 127.9; 137.5; 146.0; 146.4; 168.5; 169.7 ppm. MS: *m/z* 232 (M^+^, 100%), 233 (M^+^+1, 13%).

*3-Methyl-N-(4'-fluorophenyl)maleimide* (**14**). White solid. Mp: 122–125 °C. Yield: 72%. ^1^H-NMR (CDCl_3_): 2.18 (3H, d, *J* = 1.8 Hz; CH_3_); 6.48 (1H, q, *J* = 1.8 Hz, H-3); 7.09–7.19 (2H, m, H-2';6'); 7.28–7.36 (2H, m, H-3';5') ppm. ^13^C-NMR (CDCl_3_): 11.0; 115.8 (d, *J* = 22.7 Hz); 127.1 (d, *J* = 8.9 Hz); 136.6; 139.8; 145.8; 161.4 (d, *J* = 248.5 Hz); 168.6; 169.5 ppm. MS: *m/z* 205 (M^+^, 100%), 206 (M^+^+1, 12%).

*3-Methyl-N-(4'-chlorophenyl)maleimide* (**15**). White solid. Mp: 127–130 °C. Yield: 46%. ^1^H-NMR (CDCl_3_): 2.15 (3H, d, *J* = 1.3 Hz; CH_3_); 7.01 (1H, d, *J* = 1.6 Hz, H-3); 7.26 (2H, d, *J* = 8.8 Hz, H-3',5'); 7.44 (2H, d, *J* = 8.7 Hz, H-2',6') ppm. ^13^C-NMR (CDCl_3_): 11.0; 126.2; 129.1; 136.4; 139.6; 142.3; 150.0; 168.4; 180.7 ppm. MS: *m/z* 221 (M^+^, 100%); 223 (M^+^+2, 32%); 222 (M^+^+1, 12%).

*3-Methyl-N-(4'-bromophenyl)maleimide* (**16**). White solid. Mp: 142–144 °C. Yield: 56%. ^1^H-NMR (CDCl_3_): 2.17 (3H, d, *J* = 1.8 Hz, CH_3_); 6.48 (1H, q, *J* = 1.8Hz, H-3); 7.23–7.29 (2H, d, *J* = 8.8 Hz, 3',5'-H); 7.53–7.61 (2H, d, *J* = 8.8 Hz, 2'-6'-H) ppm. ^13^C-NMR (CDCl_3_): 11.2; 121.2; 127.2; 127.6; 130.4; 132.2; 145.9; 169.1; 170.6 ppm. MS: *m/z* 265 (M^+^, 100%), 267 (M^+^+2, 97%), 265 (M^+^+1, 12%), 268 (M^+^+3, 11%).

*3-Methyl-N-(2'-methylphenyl)maleimide* (**17**). White solid. Mp: 55–57 °C. Yield: 52%. ^1^H-NMR (CDCl_3_): 2.15 (3H, d, *J* = 1.8Hz, CH_3_); 2.16 (3H, s, CH_3_); 6.47 (1H, q, *J* = 1.8 Hz, H-3); 7.10 (1H, d, *J* = 7.3, H-3'); 7.23–7.35 (3H, m, H-4'-6') ppm. ^13^C-NMR (CDCl_3_): 11.1; 17.9; 126.8; 127.7; 128.7; 129.5; 130.6; 131.1; 136.6; 146.2; 169.7; 170.7 ppm. MS: *m/z* 203 (M^+^, 100%), 208 (M^+^+1, 13%). 

*3-Methyl-N-(2',4'-difluorophenyl)maleimide* (**18**). White solid. Mp: 80-82 °C. Yield: 85%. ^1^H-NMR (CDCl_3_): 2.01 (3H, d, *J* = 1.8Hz, CH_3_); 6.40 (1H, d, *J* = 1.8Hz, H-3); 6.89 (2H, q, *J* = 9.3 Hz; 5',6'-H); 7.15 (1H, dt, *J* = 8.6Hz; 3'-H) ppm. ^13^C-NMR (CDCl_3_): 10.8; 104.93 (dd, *J* = 26.38, 23.90 Hz); 111.78 (dd, *J* = 22.62, 3.66 Hz); 115.66 (dd, *J* = 13.43, 3.89 Hz); 127.8; 130.8 (dd, *J* = 10.09, 1.35 Hz); 146.4; 158.1 (dd, *J* = 254.42, 12.75 Hz); 162.5 (dd, *J* = 250.8, 11.3 Hz); 168.5; 169.7 ppm. MS: *m/z* 223 (M^+^, 100%), 224 (M^+^+1, 12%). 

*Rac-3-methyl-N-arylsuccinimides* **3**–**10**. Each maleimide **11**–**18** (2 mmol) was dissolved in CH_2_Cl_2_ (2 mL) to which a catalytic amount of 5% Pd/C was added. Then, the mixture was exposed to a H_2_ atmosphere at room temperature for 2 h. The crude mixture was filtered and the solvent was evaporated. The resulting product was purified by silica gel column chromatography using a mixture of hexane and ethyl acetate (9:1) as eluent. 

*(3R)-(+)-Methyl-N-arylsuccinimides* **3**–**10**. A mixture of (*R*)-(+)-methylsuccinic acid **19** (5.0 mmol in water) and substituted anilines **20**–**27** were maintained at 170 °C for 2 h and then cooled to 20 °C. The aqueous solution was extracted with Et_2_O, dried over Na_2_SO_4_, filtered, and the solvent evaporated. The mixtures were subjected to silica gel column chromatography using a mixture of hexane and ethyl acetate (9:1) as eluent. Compounds **6**, **9** and **10** (*rac* or *R*) are not described in the literature. Spectroscopic NMR data of compound **4** was identical to that previously reported [18]. Compounds *rac*-**3**, *rac*-**5**, *rac*-**7**, *rac*-**8 ** have been previously reported by Chemical Abstract Service (CAS) (**3**: CAS 105909-89-5, **5**: CAS 33624-30-5, **7**: CAS 25998-50-9, **8**: CAS 134939-24-5) but, to the best of our knowledge, complete NMR spectral data were not available in the literature, so the complete spectroscopic data of these compounds are described below. 

*(3R)-(+)-Methyl-N-(4'-methylphenyl)succinimide* (**3**), White solid. Mp: 113–115 °C. Yield: 58%. ^1^H-NMR (CDCl_3_): 1.45 (3H, d, *J* = 7.2 Hz, CH_3_); 2.38 (3H, s, CH_3_); 2.49 (1H, dd, *J* = 17.1; 3.8 Hz, H-3b); 2.94–3.16 (2H, m, H-2;3a); 7.15 (2H, d, *J* = 8.4Hz, H-2';6'); 7.27 (2H, d, *J* = 8.5 Hz, H-3';5') ppm. ^13^C-NMR (CDCl_3_): 16.9; 21.2; 126.2; 129.3; 129.8; 138.7; 175.6; 179.7 ppm. MS: *m/z* 203 (M^+^, 100%), 204 (M^+^+1, 13%). ${\left[\alpha \right]}_{D}^{27}$ +5.2 ± 1.2 (c 0.76, CHCl_3_).

*(3R)-(+)-Methyl -N-(4'-methoxyphenyl)succinimide* (**4**). White solid. Mp: 110–112 °C. Yield: 65%. ^1^H-NMR (CDCl_3_): 1.44 (3H, d, *J* = 7.2 Hz, CH_3_); 2.48 (1H, dd, *J* = 17.1; 3.8 Hz, H-3b); 2.94–3.15 (2H, m, H-2 and H-3a); 3.82 (3H, s, OCH_3_); 6.98 (2H, d, *J* = 8.4 Hz, H-2';6'); 7.19 (2H, d, *J* = 8.5 Hz, H-3';5') ppm. ^13^C-NMR (CDCl_3_): 16.9; 34.8; 36.6; 55.5; 114.5; 124.6; 127.6; 159.5;175.7; 179.8 ppm. MS: *m/z* 219 (M^+^, 100%), 220 (M^+^+1, 13%). ${\left[\alpha \right]}_{D}^{27}$ +4.2 ± 1.1 (c 0.96, CHCl_3_)

*(3R)-(+)-Methyl-N-(4'-nitrophenyl)succinimide* (**5**. White solid. Mp: 111–112 °C. Yield: 72%. ^1^H-NMR (CDCl_3_): 1.46 (3H, d, *J* = 7.2 Hz, CH_3_); 2.56 (1H, dd, *J* = 17.1; 3.9 Hz, H-3b); 2.98–3.18 (2H, m, H-2; 3a); 7.61 (2H, d, *J* = 9.3 Hz, H-2',6'); 8.34 (2H, d, *J* = 9.4 Hz, H-3',5') ppm. ^13^C-NMR (CDCl_3_): 16.9; 34.9; 36.6; 123.1; 124.4; 126.8; 137.5; 174.5; 178.6; ppm. MS: *m/z* 234 (M^+^, 100%), 235 (M^+^+1, 12%). ${\left[\alpha \right]}_{D}^{27}$ +6.1 ± 0.9 (c 0.86, CHCl_3_)

*(3R)-(+)-Methyl-N-(4'-fluorophenyl)succinimide* (**6**). White solid. Mp: 105–107 °C. Yield: 76%. ^1^H-NMR (CDCl_3_): 1.45 (3H, d, *J* = 7.2 Hz, CH_3_); 2.50 (1H, dd, 17.1; 3.8 Hz, H-3b); 2.98–3.19 (2H, m, H-2 and H-3a); 7.15 (2H, t, *J* = 8.7 Hz, H-2',6'); 7.28 (2H, dd, *J* = 8.0, 5.8 Hz, H-3',5') ppm. ^13^C-NMR (CDCl_3_): 16.9; 34.8; 36.6; 116.2 (d, *J*_C-F_ = 22.9 Hz); 128.2 (d, JC-F = 8.8 Hz); 148.5; 162.3 (d, *J*_C-F_ = 248.7 Hz); 175.3; 179.4 ppm. MS: *m/z* 207 (M^+^, 100%), 250 (M^+^+1, 12%). ${\left[\alpha \right]}_{D}^{27}$ +4.9 ± 0.8 (c 0.72, CHCl_3_).

*(3R)-(+)-Methyl-N-(4'-chlorophenyl)succinimide* (**7**). White solid. Mp: 112–113 °C. Yield: 68%. ^1^H-NMR (CDCl_3_): 1.46 (3H, d, *J* = 7.2 Hz, CH_3_); 2.51 (1H, dd, *J* = 17.1; 3.8 Hz, H-3b); 2.96–3.16 (2H, m, H-2 and H-3a); 7.26 (2H, d, *J* = 8.8 Hz, H-3',5'); 7.44 (2H, d, *J* = 8.7 Hz, H-2',6') ppm. ^13^C-NMR (CDCl_3_): 16.9; 34.8; 36.6; 126.2; 129.1; 136.4; 142.3; 178.4; 178.7 ppm. MS: *m/z* 223 (M^+^, 100%), 225 (M^+^+2, 32%), 224 (M^+^+1, 12%). ${\left[\alpha \right]}_{D}^{27}$ +4.5 ± 1.1 (c 0.66, CHCl_3_). ${\left[\alpha \right]}_{D}^{27}$ +4.5 ± 1.1 (c 0.66, CHCl_3_).

*(3R)-(+)-Methyl-N-(4'-bromophenyl)succinimide* (**8**). White solid. Mp: 134-136 °C. Yield: 49%. ^1^H-NMR (CDCl_3_): 1.46 (3H, d, *J* = 7.2 Hz, CH_3_); 2.51 (1H, dd, 17.1; 3.8 Hz, H-3b); 2.96–3.16 (2H, m, H-2 and H-3a); 7.19 (2H, d, *J* = 8.8 Hz, H-3',5'); 7.56 (2H, d, *J* = 8.7 Hz, H-2',6') ppm. ^13^C-NMR (CDCl_3_): 16.8; 34.9; 36.6; 122.4; 127.9;130.9;132.3; 174.8; 179.2 ppm. MS: *m/z* 267 (M^+^, 100%), 269 (M^+^+2, 98%), 268 (M^+^+1, 12%). ${\left[\alpha \right]}_{D}^{27}$ +5.6 ± 1.3 (c 0.58, CHCl_3_).

*(3R)-(+)-Methyl-(2'-methylphenyl)succinimide* (**9**). White solid. Mp: 102–105 °C. Yield: 74%. ^1^H-NMR (CDCl_3_): 1.46 (3H, dd, *J* = 7.15, 1.46 Hz, CH_3_); 2.13 (3H, d, *J* = 1.87 Hz, CH_3_); 2.52 (1H, ddd, *J* = 16.7, 3.4, 1.6 Hz, H-3b); 2.96–3.17 (2H, m, H- 2;3a); 7.05 (1H, dd, *J* = 7.2, 1.1 Hz; H-4'); 7.24–7.38 (3H, m, H-3';5';6') ppm. ^13^C-NMR (CDCl_3_): 16.9; 17.5; 35.2; 36.8; 126.9; 127.9; 129.5; 131.0; 131.2; 135.5; 175.4; i179.6 ppm. MS: *m/z* 201 (M^+^, 100%), 202 (M^+^+1, 13%). ${\left[\alpha \right]}_{D}^{27}$ +7.3 ± 1.7 (c 0.47, CHCl_3_)

*(3R)-(+)-Methyl-N-(2',4'-difluorophenyl)succinimide* (**10**). White solid. Mp: 102–104 °C. Yield: 49%. ^1^H-NMR (CDCl_3_): 1.45 (3H, dd, *J* = 7.15, 1.46 Hz, CH_3_); 2.52 (1H, dd, *J* = 3.4, 1.6 Hz, H-3b); 2.95–3.16 (2H, m, H-2;3a); 6.79 (2H, q, *J* = 9.3 Hz; 5',6'-H); 7.15 (1H, dt, *J* = 8.6 Hz; 3'-H) ppm. ^13^C-NMR (CDCl_3_): 16.8; 35.2; 36.7; 105.3 (dd, *J* = 26.4, 23.5 Hz), 112.0 (dd, *J* = 22.6, 3.6 Hz), 116.08 (dd, *J* = 13.4, 3.9 Hz), 130.24 (dd, *J* = 10.2, 1.7 Hz), 157.7 (dd, *J* = 255.1, 12.8 Hz), 163.1 (dd, *J* = 251.9, 11.4 Hz), 168.7; 169.2 ppm. MS: *m/z* 225 (M^+^, 100%), 226 (M^+^+1, 12%). ${\left[\alpha \right]}_{D}^{27}$ +5.3 ± 1.4 (c 0.81, CHCl_3_).

### 3.3. Biotransformations

*A. fumigatus* ATCC 26934 was grown on a plate with agarized Czapek culture medium for 3 days at 30 °C until well sporulated. 2-L Erlenmeyer flasks containing Czapek broth medium (1 L) were inoculated with suspensions of conidia (2–5 × 10^6^ CFU mL^−1^) and incubated at 30 °C for 72 h at 150 rpm on an orbital shaker (Innova 4000, New Brunswick Scientific Inc., Edison, NJ, USA).

Substrates (125 mg) in DMSO (5 mL) were poured into flasks containing the fungal biomass and the reaction mixtures were incubated at 30 °C for 72 h on an orbital shaker (150 rpm). Culture controls consisted of microorganism blank in which *A. fumigatus* ATCC 26934 was grown without substrate but fed with the same amount of DMSO. Substrate controls contained the sterile medium with the same amount of substrate and were incubated under the above conditions.

After incubation the mixtures were filtered, the aqueous phases were combined and extracted with ethyl acetate (3 × 250 mL) and the organic phases were dried over Na_2_SO_4_. Conversion percentages were determined by GC analysis of the crude extracts and determined by using the TIC (total ion current) with the following equation: % conversion: product TIC/(product TIC + substrate TIC) × 100. Compounds were purified by silica gel column chromatography using a mixture of hexane and ethyl acetate (9:1) as eluent.

### 3.4. Antifungal Susceptibility Testing

The test fungi belonged either to the American Type Culture Collection (ATCC, Rockville, MD, USA), or the Culture Collection of CEREMIC (Centro de Referencia en Micología-CCC, Facultad de Ciencias Bioquímicas y Farmacéuticas, Rosario, Argentina). Species of *Candida* genus: *C. albicans* ATCC 10231, *C. tropicalis* CCC 131-2000, *C. krusei* CCC 117-2000, *C. glabrata* CCC 115-2000, *C. parapsilosis* CCC 124-2000, *C. lusitaniae* CCC 134-2000; *S. cerevisiae* ATCC 9763; *C. neoformans* ATCC 32264, *M. gypseum* CCC 115-2000, *T. rubrum* CCC 113-2000 and *T. mentagrophytes* ATCC 9972 were used.

Strains were grown on Sabouraud-chloramphenicol agar slants at 30 °C, maintained on slopes of Sabouraud-dextrose agar (SDA, Oxoid), and sub-cultured every 15 days to prevent pleomorphic transformations. Inocula were obtained according to reported procedures [23,24] and adjusted to 1–5 × 10^3^ colony forming units (CFU) mL^−1^.

The antifungal activity was determined by using broth microdilution techniques following the guidelines of the CLSI for yeasts and for filamentous fungi [23,24]. Compounds stock solutions were two-fold diluted from 250 to 0.24 μg ml^−1^ (final volume = 100 μL) and a final DMSO (Sigma) concentration <1% in the culture media RPMI-1640 (Sigma) buffered to pH 7.0 with MOPS (Sigma).

The test was performed in 96 wells-microplates and included: Test wells (TW), containing the compound solution and the inoculum of the fungus; Growth Control Well (GCW), that contains compound-free medium and inoculum; Blank Test Wells (BTW), with the compound, culture medium and sterile water instead of inoculum; Sterility Control Well (SCW) containing the compound diluted in culture medium and sterile water. Ketoconazole (Sigma), terbinafine (Sigma) and amphotericin B (Sigma), were used as standard controls.

Plates were incubated in a moist, dark chamber 24 h for yeasts and 5 d for dermatophytes and the MIC (Minimum Inhibitory Concentration) was determined. For yeasts, microplates turbidity due to fungal growth were determined at 405 nm in a VERSA Max microplate reader (Molecular Devices, Sunnyvale, CA, USA) and the % of growth were calculated as follows: % of growth = [(OD_TW_ − OD_BTW_)/(OD_GCW_ − OD_SCW_) ×100.

### 3.5. Statistical Tests

Multiple comparisons were performed by the non*-*parametric ANOVA, Kruskal-Wallis test followed by Dunn's multiple comparisons and Wilcoxon’s signed rank test. The associations between MIC for the different groups were corroborated with the Score’s test [25]. Individual comparisons between groups were performed with Student’s t test. A value of *p* < 0.05 was considered significant.

## 4. Conclusions

The antifungal behavior of eighteen enantiomerically pure (3*R*)-methyl- and (3*R,*4*R*)-dimethyl-*N-*phenyl-, *N*-phenylalkyl- or *N*-arylsuccinimides, obtained by a low-cost and environmentally friendly *A. fumigatus*-catalyzed reduction of maleimides, were compared to that of the corresponding racemic ones. Results showed that the biotransformation products displayed statistically significant higher antifungal activity against a panel of human opportunistic pathogenic fungi including yeasts as well as dermatophytes than racemic ones as demonstrated by different approaches. These findings can be highly useful for the development of chiral methylated succinimides as antifungal drugs by the pharmaceutical industry and in a broader sense, they open new avenues for the development of highly active drugs containing other 3- and 3,4-substituted chiral succinimides [26].
